# Male and female mice are similarly susceptible to chronic nondiscriminatory social defeat stress despite differences in attack frequency from aggressor

**DOI:** 10.1007/s00213-025-06858-z

**Published:** 2025-07-12

**Authors:** Allyson Bazer, Katherine Denney, Maria Chacona, Catherine Montgomery, Shriya Vinod, Urboshi Datta, Benjamin Adam Samuels

**Affiliations:** 1 Department of Psychology, School of Arts and Sciences, Rutgers, the State University of New Jersey, Piscataway, NJ USA; 2 Behavioral and Systems Neuroscience Graduate Program, Department of Psychology, Rutgers, the State University of New Jersey, Piscataway, NJ USA; 3 Department of Animal Sciences, School of Environmental and Biological Sciences, Rutgers, the State University of New Jersey, New Brunswick, NJ USA

**Keywords:** Chronic stress, Social defeat stress, Sex differences, Reward valuation

## Abstract

**Rationale:**

Chronic stress is a major precipitating factor for mood disorders, which are diagnosed twice as frequently in women as in men. However, most preclinical models of chronic social defeat stress have limited use in females due to reduced aggression toward female intruders. The chronic non-discriminatory social defeat stress (CNSDS) model addresses these limitations by enabling the study of stress susceptibility across sexes in a variety of behavioral tasks including avoidance and simple reward behaviors. However, the effect of CNSDS susceptibility on complex reward behaviors has yet to be studied.

**Objectives:**

Building on previous work validating CNSDS in both sexes, we aimed to refine the protocol for identifying CD-1 aggressors for CNSDS, optimize criteria for classifying susceptibility and resilience to stress, and assess how stress susceptibility affects complex reward behavior.

**Methods:**

We evaluated aggression through attack frequency and submissive posture during CD-1 aggressor screening and CNSDS sessions to establish screening parameters. Following CNSDS exposure, male and female intruder mice were tested in an operant satiety-based outcome devaluation task.

**Results:**

We observed that despite receiving fewer attacks, female mice are equally susceptible to CNSDS as males. CNSDS abolished satiety-based outcome devaluation, regardless of sex, in susceptible mice, but not in resilient mice.

**Conclusions:**

These data suggest that CNSDS-defined stress phenotypes are behaviorally relevant across sexes and extend to deficits in complex reward behaviors, supporting CNSDS as a valuable model for studying sex-specific stress outcomes in mood disorders.

## Introduction

Millions of people suffer from major depressive disorder (MDD), a mood disorder that the World Health Organization has predicted will outpace ischemic heart disease as the number one cause of disease burden by 2030 (Tucci and Moukaddam 2017). Mood disorders, such as MDD and anxiety, are often precipitated by chronic stress. Frequent stress exposure causes adaptation to cortisol release, ultimately altering the HPA axis and stress response (Nemeroff 2004; de Kloet et al. 2005; Hammen et al. 2009). Therefore, while complex mood disorders diagnosed with self-report questionnaires (such as MDD) cannot be directly modeled in rodents, chronic stress paradigms can be used to recapitulate some behavioral phenotypes. However, common chronic social stress paradigms used in mice, such as classic forms of chronic social defeat stress, were developed for males and are not as effective in females. Other stress paradigms, such as chronic corticosterone administration, have inconsistent effects in female mice (Mekiri et al. 2017; Berger et al. 2019; Yohn et al. 2019a; Holloway et al. 2023). Because of this, most historic pre-clinical chronic stress paradigms exclude females, despite MDD diagnoses occurring twice as frequently in women as in men (Kessler 2003; Kessler et al. 2005; Beery and Zucker 2011). Given the prevalence of MDD in women, there is a need for increased use of female mice in preclinical research. Our lab previously reported a variation of chronic social defeat stress that permits inclusion of female mice (Yohn et al. 2019b).

Chronic social defeat stress (CSDS) is a paradigm in which experimental intruder mice experience repeated stressful bouts of aggression from a resident CD-1 aggressor mouse. Following these defeat sessions, a social interaction test is used to define susceptible (SUS) mice, which display behavioral and neural changes consistent with increased social avoidance, and resilient (RES) mice, which do not show these changes (Krishnan et al. 2007; Golden et al. 2011). Social interaction test-defined SUS and RES phenotypes following social defeat stress procedures are correlated with changes in sucrose preference and avoidance behaviors (Venzala et al. 2012; Macedo et al. 2018; Yohn et al. 2019b; Guo et al. 2020). However, surprisingly few studies have asked how chronic social defeat stress affects more complex reward-related behaviors, such as reward valuation, especially considering dysfunctional reward processing is commonly found in mood disorders and frequently persists after treatment (Admon and Pizzagalli 2015). Furthermore, it is unknown whether SUS and RES phenotypes have any correlation with stress-induced changes in reward valuation behaviors.

CSDS exploits male rodent territorial aggression. While this behavior is natural in male mice, most strains of female mice are less aggressive, and aggressive male mice tend to mount rather than attack females (Debold and Miczek 1984; Lopez and Bagot 2021). Female resident aggressor mice do not produce the necessary bouts of attack characterizing CSDS unless co-housed with a castrated male (Newman et al. 2019), as female-female aggression in mice is limited to specific territorial species (Trainor et al. 2011; Smith et al. 2013), maternal aggression (Bosch and Neumann 2010; Bourke and Neigh 2012), and following ovariectomy (Debold and Miczek 1984). There are chronic stress paradigms that work predominately in female mice, such as witness defeat stress and social instability stress; however, these stress paradigms are not as effective in male mice and make direct comparisons between sexes challenging (Haller et al. 1999; Sial et al. 2016; Yohn et al. 2019a; Schuler et al. 2024). Importantly, over the last decade several successful variations of classic social defeat stress were designed to allow inclusion of female mice. These include targeted chemogenetic activation of ventromedial hypothalamus and daily urine applications to increase aggression toward female mice (Takahashi et al. 2017; Harris et al. 2018). In addition, we developed chronic non-discriminatory social defeat stress (CNSDS), which simultaneously exposes a male and female C57BL/6J to an aggressor and is effective in both sexes (Yohn et al. 2019b). CNSDS potentially provides the advantage of males and females having a shared and similar experience during defeat sessions, which could make direct comparisons between sexes more meaningful.

We previously demonstrated that CNSDS yields aggression toward both male and female intruder mice, which resulted in behavioral and neuroendocrine responses consistent with chronic stress exposure. Importantly, we also found that CD-1 aggressor mice attack female intruders far less than male intruders, which has been replicated in many other studies that have utilized or adapted the CNSDS protocol (Yohn et al. 2019b; Zhu et al. 2023; Smith et al. 2023; Bennett et al. 2024). In addition, some labs report that they did not observe increased social avoidance, or susceptibility, in female mice (Parel et al. 2023), which suggests that female mice may display susceptibility and response to chronic stress differently than male mice (Willmore et al. 2022; Balouek et al. 2023; Schuler et al. 2024). Despite these drawbacks, many studies have used CNSDS as an effective paradigm to investigate behavioral and physiological effects of chronic stress in female mice (Yohn et al. 2019b; Dieterich et al. 2021; Zhu et al. 2023; Balouek et al. 2023; Smith et al. 2023; Bennett et al. 2024). In this study, one aim is to update the CNSDS procedure to better facilitate the inclusion of female mice in chronic stress research.

We also previously reported that CNSDS can induce a change in outcome devaluation behavior (Dieterich et al. 2021). Outcome devaluation is a reward valuation task that involves satiety-based devaluation of a food reward during an instrumental operant conditioning task. However, we did not assess SUS and RES phenotypes in that previous study. Therefore, a second aim of the study was to determine whether CNSDS-induced SUS and RES behavioral phenotypes extend to outcome devaluation, which is a translationally relevant complex reward valuation behavior (Der-Avakian et al. 2016).

Here, we introduce an improved screening protocol to identify and rank CD-1 aggressors for CNSDS. We also introduce a new SIT ratio calculation that categorizes susceptibility to CNSDS by directly assessing social avoidance to the CD-1 aggressor rather than general social avoidance, and show that despite receiving fewer attacks, female mice are equally susceptible to CNSDS as male mice. Finally, we investigate the effect of CNSDS on satiety-based outcome devaluation and observe a novel effect of stress susceptibility on sensitivity to a devalued outcome.

## Methods

### Subjects

Eight-week-old male and female C57BL/6J mice (*n* = 52) were purchased from Jackson Laboratories (Bar Harbor, ME) and retired breeder male CD-1 mice (*n* = 70) were purchased from Charles River Laboratories. Mice were maintained on a 12L:12D schedule with lights turning on at 6 a.m. and turning off at 6 p.m. Throughout the CNSDS procedure, all food and water were provided ad libitum. During the outcome devaluation procedure, mice were food restricted to 85% of their body weight. Behavioral testing took place in the morning, between the hours of 8 a.m. and 1 p.m. All experiments were conducted in compliance with NIH laboratory animal care guidelines and approved by Rutgers University Institutional Animal Care and Use Committee (IACUC).

### CD-1 Aggressor Screening

**Fig. 1:**
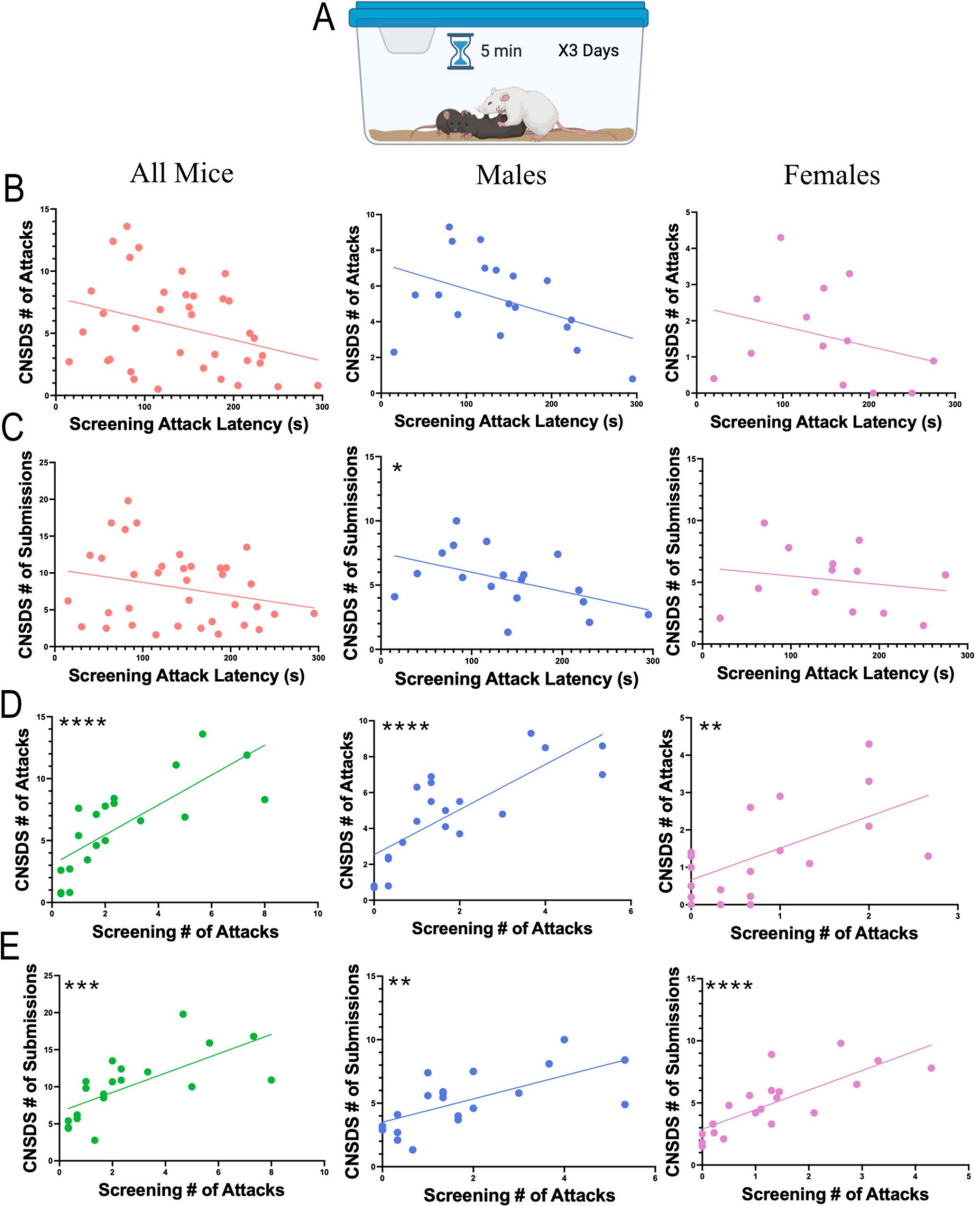
Comparison of CD-1 aggression during screening and CNSDS defeat sessions. (**A**) During screening, CD-1 aggressors are exposed to a male and female C57BL/6J intruder for a 5-minute session for three consecutive days. During this time, number of attacks, latency to first attack, number of submissions, and number of mountings are recorded. (**B**) Linear regressions showing the correlation between the average latency to attack during screening and the average number of attacks per CNSDS session by CD-1 in all mice (R^2^ = 0.1043, F(1,34) = 3.957, *p* = 0.0548), males (R^2^ = 0.1954, F(1,16) = 3.886, *p* = 0.0662), and females (R^2^ = 0.08856, F(1,11) = 1.069, *p* = 0.3234). (**C**) Linear regressions showing the correlation between the average latency to attack during screening and the average number of submissions received per CNSDS session by CD-1 in all mice (R^2^ = 0.06308, F(1,34) = 2.289, *p* = 0.1395), males (R^2^ = 0.2291, F(1,16) = 4.755, *p* = 0.0445), and females (R^2^ = 0.03748, F(1,11) = 0.4284, *p* = 0.5262). (**D**) Linear regressions showing the correlation between the average number of attacks during screening and the average number of attacks per CNSDS session by CD-1 in all mice (R^2^ = 0.5896, F(1,18) = 25.86, *p* < 0.0001), males (R^2^ = 0.6249, F(1,18) = 29.99, *p* < 0.0001), and females (R^2^ = 0.3496, F(1,18) = 9.674, *p* = 0.0060). (**E**) Linear regressions showing the correlation between the average number of attacks during screening and the average number of submissions received per CNSDS session by CD-1 in all mice (R^2^ = 0.4741, F(1,18) = 16.23, *p* = 0.0008), males (R^2^ = 0.4355, F(1,18) = 13.89, *p* = 0.0015), and females (R^2^ = 0.5971, F(1,18) = 26.67, *p* < 0.0001). *p < 0.05, **p < 0.01, ***p < 0.001, ****p < 0.0001

Retired CD-1 breeder adult males (*n* = 70) were purchased from Charles River Laboratory (Wilmington, MA). To screen for aggressive behavior, screener (non-experimental) female and male C57BL/6J mice aged 8–20 weeks were simultaneously placed in the home cage of a CD-1 mouse (Fig. 1A). Latency to attack (in seconds), the number of attacks against male and female intruder mice, the number of bouts of submissive posture to the CD-1 aggressor, and the number of times the CD-1 mounted the intruder mice were recorded. CD-1 mice that attacked the female screener mice within the 5-minute screening sessions were selected for further analysis. An attack was defined as a bite or scratch to the intruder mouse and often included wrestling behavior. CD-1 mice were then ranked by average number of attacks across the three days of screening, and CD-1 mice with the highest average attacks (an average of at least 1 attack per screening session was used as a minimum threshold) were chosen as aggressors. Screening sessions were scored live and manually. Sessions were also video recorded as a backup to allow the observer to confirm the live scoring.

### Chronic non-discriminatory social defeat stress

**Fig. 2:**
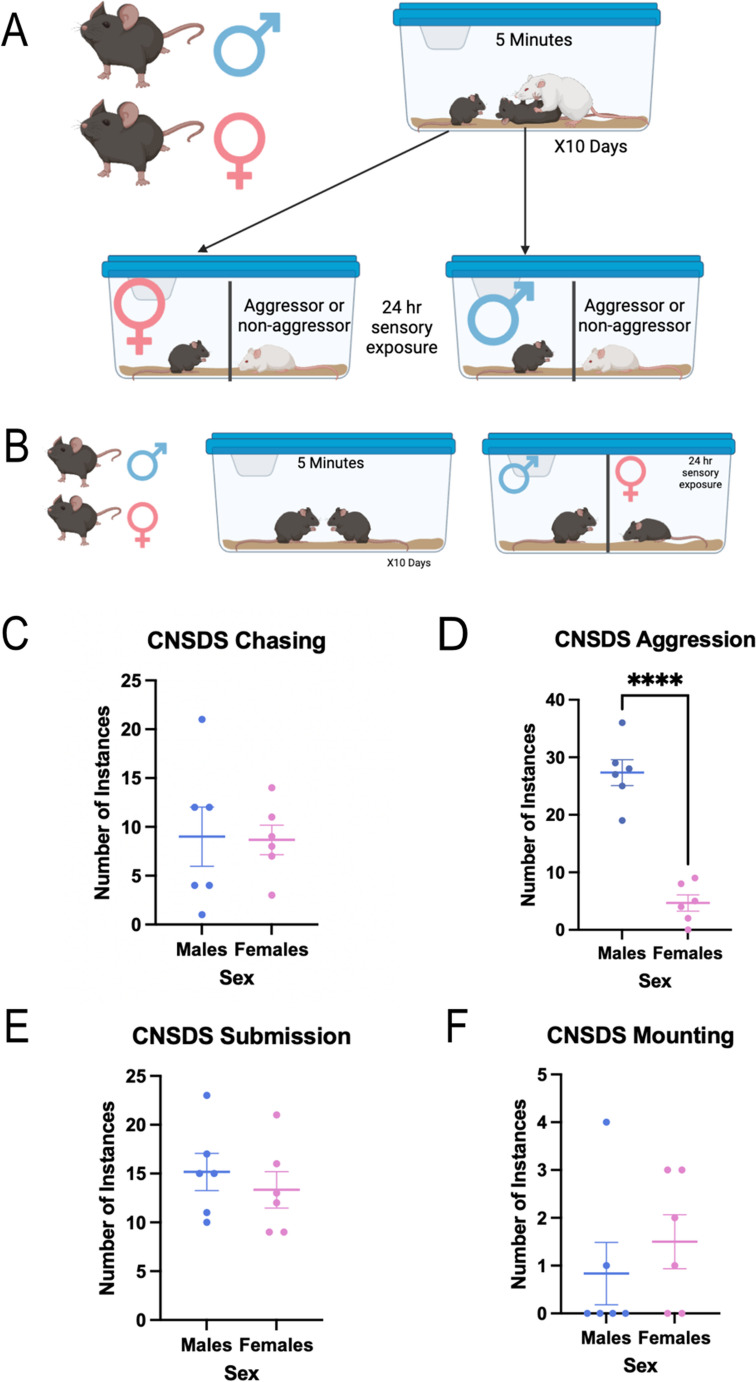
Male intruder mice are attacked significantly more than female mice, but male and female mice show equal instances of submission. (**A**) The male and female C57BL/6J pairs were placed into the home cage of novel CD-1 aggressors for 10 consecutive daily 5-min sessions. (**B**) Control C57BL/6J mice were allowed to interact for 5 min in the absence of an aggressor CD-1 male over the 10 days and were co-housed on either side of a perforated, plexiglass divider. (**C**) There was no significant difference between the number of occurrences of chasing male and female intruders by CD-1 aggressors (t(10) = 0.09825, *p* = 0.9237). (D) CD-1 aggressor mice attacked male intruders significantly more than female intruders (t(10) = 8.513, *p* < 0.0001). (**E**) There was no significant difference between the number of occurrences of male and female submission to CD-1 aggressors (t(10) = 0.6862, *p* = 0.5082). (F) There was no significant difference between the number of occurrences of mounting male and female intruders by CD-1 aggressors (t(10) = 0.7727, *p* = 0.4576). ****p<0.0001

Adult male and female C57BL/6J mice were randomly assigned to either CNSDS or no stress control groups. During the experimental phase, male C57BL/6J mice were matched with the same female C57BL/6J mice throughout the social defeat. The male and female C57BL/6J pairs were placed into the home cage of a novel CD-1 aggressor for 10 consecutive daily 5-min sessions (Fig. 2A). C57BL/6J pairs were shifted daily so CD-1 aggressors did not interact with the same intruder pair twice. On alternating days, either the male or female C57BL/6J mouse was co-housed with the CD-1 aggressor with which they had interacted, while the other was co-housed with a novel CD-1. Co-housed subjects were separated by clear, perforated plexiglass barriers that permitted sensory, but not physical, interaction. In total, both C57BL/6J male and female mice experienced 5 days of sensory exposure to an aggressor CD-1 they had just interacted with and 5 days of sensory exposure to a different CD-1. During the 5-min sessions, attack latency and frequency, submission behavior, and mounting behavior were measured and recorded for each of the 10 days. Submission behavior was defined as a defensive, upright posture where the stomach was exposed, and the forepaws were stretched forward. The no stress control group of female and male C57BL/6J mice were placed into a cage and allowed to interact for 5 min in the absence of an aggressor CD-1 male over the 10 days. These opposite sex control mice were housed on either side of a perforated, plexiglass divider and placed on a separate rack from chronic stress mice for 24 h until the next interaction session (Fig. 2B). CNSDS sessions were scored live and manually. Sessions were also video recorded as a backup to allow the observer to confirm the live scoring.

### Behavioral testing

#### Social interaction test

**Fig. 3:**
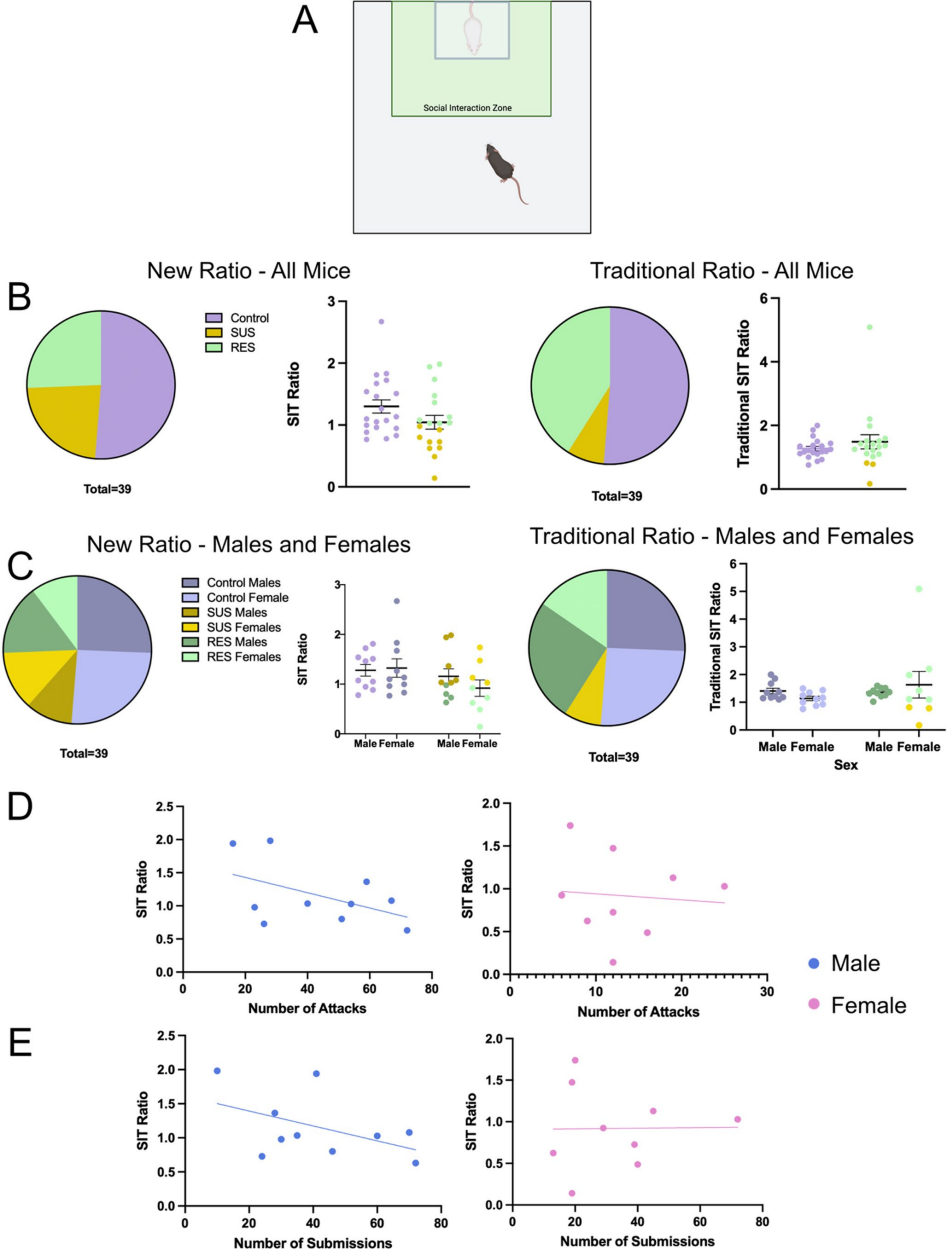
CNSDS results in both susceptible and resilient mice regardless of sex. (**A**) The social interaction test (SIT) was used to analyze social interaction and avoidance behavior in the presence of a novel CD-1 mouse. It consists of 3 2.5-minute trials, the first of which has no mouse present within the social interaction zone, the second has a novel CD-1, and the third has a C57BL/6J of the opposite sex. (**B**) Split of control, SUS, and RES mice after CNSDS using the new SIT ratio (time spent in presence of CD1/time spent in presence of C57) and traditional SIT ratio (time spent in presence of CD1/time spent in absence of all mice). (**C**) Split of control, SUS, and RES mice separated by sex using the new SIT ratio and traditional SIT ratio. (**D**) Linear regressions showing the correlation between the number of attacks received and SIT ratio in males (R^2^ = 0.2336, F(1,8) = 2.438, *p* = 0.1571) and females (R^2^ = 0.0074, F(1,7) = 0.0522, *p* = 0.8258). (**E**) Linear regressions showing the correlation between the number of instances of submissive posture and SIT ratio in males (R^2^ = 0.2252, F(1,8) = 2.326, *p* = 0.1658) and females (R^2^ = 0.0002, F(1,7) = 0.0013, *p* = 0.9718)

To assess susceptibility and resilience status, we ran a social interaction test (SIT) in the presence of a novel CD-1 aggressor and a novel C57BL6/J mouse. Mice were placed in a plexiglass open field arena (43 cm x 43 cm) for three consecutive 2.5-min trials. The first trial had no mouse present in a perforated Plexiglass container within the social interaction zone (14 cm x 24 cm). The second trial had a novel CD-1 placed in the perforated container within the social interaction zone (Fig. 3A). The third trial had a novel C57BL/6J mouse of the opposite sex within the social interaction zone. Overhead cameras recorded behavior, and Bonsai software (Lopes et al. 2015) measured time spent in the pre-defined interaction zone. Social interaction behavior was measured by time spent in the interaction zone during the second (CD-1) and third (C57) trial, with a social interaction ratio calculated: (interaction time with CD-1 present)/(interaction time with opposite sex C57 present). Mice with an interaction ratio < 1 were categorized susceptible (SUS), while mice with an interaction ratio > 1 were categorized as resilient (RES) to CNSDS. For comparison, social avoidance behavior was also measured by time spent in the interaction zone during the second (CD-1) and first (no mouse) trials, with a traditional social interaction ratio calculated: (interaction time with CD-1 present)/(interaction time with no mouse present).

#### Outcome devaluation

Male and female C57BL/6J experimental mice were trained to lever press and then tested in standard mouse instrumental conditioning chambers (Med Associates, Fairfax, VT), connected via a power control and interface unit to a dedicated computer with MED-PC IV software (Med Associates, Fairfax, VT) running custom codes. Each instrumental chamber consisted of a single retractable response lever with a reward port delivering 20 mg dustless precision food pellet reinforcers (Bio-Serv, Flemington, NJ), connected by Y-tubing to pellet hoppers. CNSDS-exposed males (*n* = 9) and females (*n* = 10) were first exposed to instrumental responding on a Fixed Ratio 1 (FR1) schedule of reinforcement, where each lever press is reinforced by a sucrose pellet. A single lever was ejected into the chamber at the start of each trial, and was retracted following a lever press, coinciding with a single reward pellet being delivered into the reward port for consumption. The lever reappeared after five seconds following a successful trial. Once mice reached the threshold of 20 lever presses per session, they then completed a Variable Ratio 2 (VR2) session, where every 1, 2, or 3 lever presses was reinforced by a sucrose pellet. Once all mice had lever pressed at least 20 times in an FR1 and VR2 session, they progressed to outcome devaluation testing (*n* = 13). Mice that did not reach or surpass the threshold of 20 lever presses were excluded (*n* = 6).

**Fig. 4:**
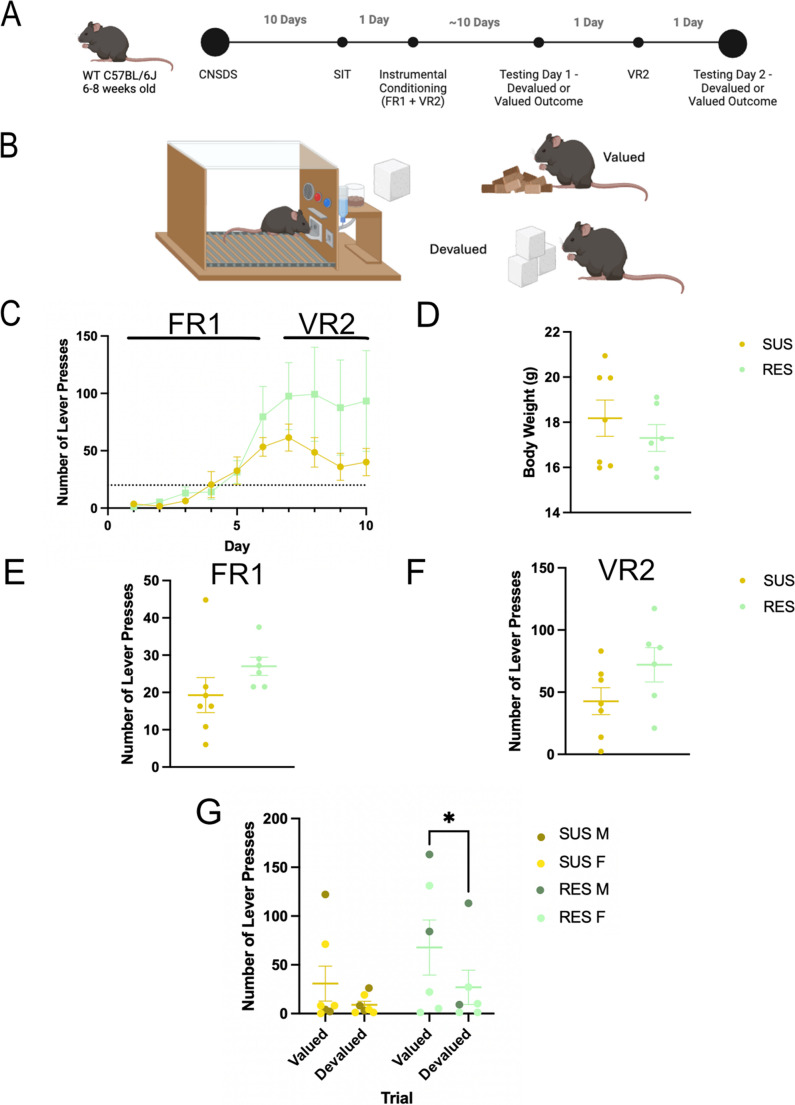
Sensitivity to outcome devaluation is influenced by susceptibility to CNSDS. (**A**) Timeline of CNSDS, operant training, and counterbalanced outcome devaluation testing. (**B**) Mice were pre-fed with either standard lab chow or reinforcer pellets one hour before a 5-minute extinction operant test. (**C**) Training data for the operant task on an FR1 or VR2 schedule. Days 1–6 are FR1, and Days 7–10 are VR2. The dotted line denotes the threshold of 20 lever presses. A 2-way RM ANOVA showed an effect of training day (F(9,108) = 8.633, *p* = 0.0024), but not of susceptibility (F(1,11) = 2.056, *p* = 0.1771) or an interaction between training day and susceptibility (F (9, 108) = 1.320, *p* = 0.2348) on average number of lever presses across the ten days. Planned multiple comparisons for each day show that RES mice do not press significantly more on training days than SUS mice. (**D**) There was no significant difference in average body weight between SUS and RES mice (t(11) = 0.8523, *p* = 0.4122). (**E**) There was no significant difference in number of lever presses during FR1 training between SUS and RES mice (t(11) = 1.386, *p* = 0.1931). (**F**) There was no significant difference in number of lever presses during VR2 training between SUS and RES mice (t(11) = 1.694, *p* = 0.1183). (**G**) RES mice are more sensitive to satiety-based outcome devaluation than SUS mice. A 2-way RM ANOVA showed an effect of trial (F(1,11) = 7.264, *p* = 0.0208), but not susceptibility F(1,11) = 1.398, *p* = 0.2620) or an interaction between trial and susceptibility (F(1,11) = 0.6006, *p* = 0.4547) on number of lever presses in outcome devaluation. Planned multiple comparisons to assess sensitivity to devaluation in RES and SUS mice show that RES mice press significantly more during the valued trial than the devalued trial (*p* = 0.0375) while SUS mice do not (*p* = 0.1853). *p < 0.05

Mice then completed a satiety-based outcome devaluation procedure. Valued and devalued sessions were counterbalanced across two days of testing, separated by a single VR2 session day to re-acquire the response-reward relationship. In the devalued session, mice were pre-fed with reinforcer pellets in the home cage 1 hour before a 5-minute extinction test where the lever was ejected and responses were recorded, but no reinforcers were delivered. In the valued session, mice were pre-fed with standard lab chow in the home cage 1 hour before an identical 5-minute extinction test (Fig. 4B). Devalued and valued sessions were counterbalanced between groups, with half of the mice completing the valued session first and the devalued session second, and the other half completing the devalued session first and the valued session second. Response in “devalued’ and “valued” sessions were measured and compared to determine the effect of CNSDS on responding in both sessions.

### Statistical analyses

To investigate the correlation of attack behavior (attack latency and attack frequency) during screening to attack behavior (attack frequency) during CNSDS defeat sessions, a linear regression was used. To assess sex differences in chasing, submission, attack, and mounting frequency, independent samples t-tests were used. To assess the distribution of stress susceptibility compared to the expected outcome, binomial tests were run. To investigate the effect of number of attacks and number of instances of submissive posture on susceptibility to stress, a linear regression was used. To assess the effect of stress susceptibility on outcome devaluation training, a 2 × 2 repeated measures ANOVA with planned Fisher’s LSD comparison test was used. To assess differences between SUS and RES mice in body weight and average number of lever presses during FR1 and VR2 schedule training, independent samples t-tests were used. To assess the effect of stress susceptibility on sensitivity to outcome devaluation using the new SIT ratio, a repeated measures 2 × 2 ANOVA with planned Fisher’s LSD comparisons was performed. GraphPad Prism 10 was used for all analyses.

## Results

### Number of attacks during screening, but not latency to attack, correlates with number of attacks during CNSDS in male and female mice

Published screening parameters for CNSDS and other social defeat protocols state that the aggressor must attack the intruder mice within 60 s every day for three days, which is the common threshold in other social defeat stress protocols (Golden et al. 2011; Yohn et al. 2019b). An important difference with CNSDS is that aggressors are screened with both male and female intruder mice (Fig. 1A). However, with CNSDS screening many CD-1 mice do not attack within the first 60 s, resulting in few mice reaching the threshold to be considered an aggressor despite eventually attacking the intruder mice within the five-minute screening period. It is unclear if latency to attack during CNSDS screening is correlated with increased aggression during CNSDS defeat sessions. Therefore, we performed simple linear regressions to determine if latency to attack and number of attacks during screening significantly correlated with several measures of aggression during CNSDS defeat sessions.

The average CD-1 latency to attack during screening did not significantly correlate to number of attacks per CNSDS defeat session across all mice (R^2^ = 0.1043, F(1,34) = 3.957, *p* = 0.0548).This was consistent for both attacks against male intruders (R^2^ = 0.1954, F(1,16) = 3.886, *p* = 0.0662) and female intruders (R^2^ = 0.08856, F(1,11) = 1.069, *p* = 0.3234) (Fig. 1B). Across all mice, the average latency to attack during screening also did not significantly correlate to number of submissions received by CD-1 aggressors per CNSDS defeat session (R^2^ = 0.06308, F(1,34) = 2.289, *p* = 0.1395) (Fig. 1C). While this was consistent for submissions from female intruders (R^2^ = 0.03748, F(1,11) = 0.4284, *p* = 0.5262), the correlation between average latency to attack during screening and the number of submissions received from male intruders was significant (R^2^ = 0.2291, F(1,16) = 4.755, *p* = 0.0445) (Fig. 1C).

By contrast, the average number of attacks during screening was significantly correlated to the number of attacks per CNSDS defeat session (R^2^ = 0.5896, F(1,18) = 25.86, *p* < 0.0001), and this was consistent for attacks against male intruders (R^2^ = 0.6249, F(1,18) = 29.99, *p* < 0.0001) and female intruders (R^2^ = 0.3496, F(1,18) = 9.674, *p* = 0.0060) (Fig. 1D). The average number of attacks during screening also significantly correlated to the number of submissions received by CD-1 aggressors per CNSDS defeat session (R^2^ = 0.4741, F(1,18) = 16.23, *p* = 0.0008), and this was consistent for submissions from male (R^2^ = 0.4355, F(1,18) = 13.89, *p* = 0.0015) and female (R^2^ = 0.5971, F(1,18) = 26.67, *p* < 0.0001) intruders (Fig. 1E). These data suggest that the average number of attacks during screening is a better predictor of CD-1 aggression during CNSDS defeat sessions than latency to attack.

### Male intruder mice are attacked significantly more than female mice, but male and female mice show equal instances of submission

Based on these data, we defined aggressive mice during screening as having attacked the female and averaging at least 1 attack per day. During CNSDS, CD-1 aggressor mice chased male intruders an average of 9 times per defeat session, and chased female mice an average of 8.667 times per session (t(10) = 0.09825, *p* = 0.9237) (Fig. 2C). CD-1 aggressor mice attacked male intruders an average of 27.33 times per session, while only attacking female intruders an average of 4.667 times per session (t(10) = 8.513, *p* < 0.0001) (Fig. 2D). Male intruder mice submitted to the CD-1 aggressor an average of 15.17 times per session, while female intruder mice submitted an average of 13.33 times per session (t(10) = 0.6862, *p* = 0.5082) (Fig. 2E). Male intruder mice were mounted by the CD-1 aggressor an average of 0.8333 times per session, while female intruders were mounted an average of 1.5 times per session (t(10) = 0.7727, *p* = 0.4576) (Fig. 2F). These data show that male intruder mice are attacked significantly more than female intruder mice during CNSDS defeat sessions, while there are equal instances of submission to, chasing by, and mounting by the CD-1 aggressor in male and female intruder mice.

### CNSDS results in both susceptible and resilient mice regardless of sex

To determine if CNSDS using CD-1 aggressors screened with the new parameters would result in susceptible and resilient mice, we ran the social interaction test (SIT) to analyze social interaction and avoidance behavior in the presence of a CD-1 mouse. The SIT ratio was calculated based on the time spent in the social interaction zone when the CD-1 was present compared to a C57 mouse of the opposite sex being present. Of the 19 mice exposed to stress, 9 were susceptible (SUS) and the other 10 were resilient (RES) (Fig. 3B “New Ratio– All Mice”). A binomial test showed that the outcome of SUS vs. RES mice was not significantly different from the expected 60/40 split (*p* = 0.3490) (Golden et al. 2011). Of the 10 males exposed to stress, 4 were SUS and 6 were RES. Of the 9 females exposed to stress, 5 were SUS and 4 were RES (Fig. 3C “New Ratio – Males and Females”). The traditional SIT ratio was also calculated based on the time spent in the social interaction zone when the CD-1 was present compared to no mouse being present. Of the 19 mice exposed to stress, 3 were SUS and the other 16 were RES (Fig. 3B “Traditional Ratio – All Mice”). A binomial test showed that the outcome of SUS vs. RES mice was significantly different from the expected 60/40 split (*p* = 0.0002). Of the 10 males exposed to stress, 0 were SUS and 10 were RES. Of the 9 females exposed to stress, 3 were SUS and 6 were RES (Fig. 3C “Traditional Ratio – Males and Females”). These data confirm that CNSDS results in susceptible and resilient populations using the novel SIT ratio in both male and female C57BL/6J mice.

### Number of attacks and submissions does not influence susceptibility to stress

To determine if number of attacks received or instances of submissive posture influences susceptibility to stress, we ran linear regressions to investigate the correlation between instances of these behaviors and SIT ratio. Interestingly, the number of attacks received did not correlate to SIT ratio in male (R^2^ = 0.2336, F(1,8) = 2.438, *p* = 0.1571) or female (R^2^ = 0.0074, F(1,7) = 0.0522, *p* = 0.8258) mice (Fig. 3D). The number of instances of submissive posture also did not correlate to SIT ratio in male (R^2^ = 0.2252, F(1,8) = 2.326, *p* = 0.1658) or female (R^2^ = 0.0002, F(1,7) = 0.0013, *p* = 0.9718) mice (Fig. 3E). These data suggest that the number of attacks received by intruder mice and the number of submissions to the CD-1 aggressor are not relevant measures for determining susceptibility to stress.

### Sensitivity to outcome devaluation is influenced by susceptibility to CNSDS

We next wanted to assess whether CNSDS-induced susceptible and resilient phenotypes correlated with performance in an outcome devaluation task. We trained male and female C57BL/6J mice that had undergone CNSDS to lever press in an operant task on an FR1 and VR2 schedule for a sucrose reward. Once the mice learned the task, they were pre-fed with either lab chow (valued) or sucrose pellets (devalued) in a counterbalanced satiety-based outcome devaluation task (Fig. 4A and B). Both SUS and RES mice were able to learn the task and passed the threshold of 20 lever presses. A 2-way repeated measures ANOVA showed an effect of training day (F (9, 108) = 8.633, *p* = 0.0024), but not of susceptibility (F (1, 12) = 2.056, *p* = 0.1771), nor an interaction between training day and susceptibility (F (9, 108) = 1.320, *p* = 0.2348) on average number of lever presses across the ten-day period. RES mice do not press significantly more on training days than SUS mice in the FR1 (*p* = 0.1931) or VR2 (*p* = 0.1183) schedule (Fig. 4C, E and F). There was no significant difference in average body weight between SUS and RES mice (*p* = 0.4122; Fig. 2D).

Mice then completed a satiety-based outcome devaluation procedure. Valued and devalued sessions were counterbalanced across two days of testing, separated by a single VR2 session day to re-acquire the response-reward relationship. A 2-way repeated measures ANOVA showed an effect of trial (F(1,11) = 8.339, *p* = 0.0148), but not sex (F(1,11) = 2.171, *p* = 0.1686) or an interaction between trial and sex (F(1,11) = 0.8019, *p* = 0.3897) on number of lever presses in outcome devaluation. The groups were combined as no sex effect was observed (Shansky 2019). A 2-way repeated measures ANOVA showed an effect of trial (F(1,11) = 7.264, *p* = 0.0208), but not susceptibility F(1,11) = 1.398, *p* = 0.2620) or an interaction between trial and susceptibility (F(1,11) = 0.6006, *p* = 0.4547) on number of lever presses in outcome devaluation. We next performed planned Fisher’s LSD multiple comparisons to determine sensitivity to outcome devaluation within the SUS and RES groups. Interestingly, RES mice press significantly more during the valued trial than the devalued trial (*p* = 0.0375) while SUS mice do not press significantly more during the valued trial compared to the devalued trial (*p* = 0.1853) (Fig. 4G), demonstrating that RES mice were sensitive to satiety-based outcome devaluation while SUS mice were not.

## Discussion

This study aimed to improve the CNSDS protocol previously published by our lab (Yohn et al. 2019b). First, we updated the CD-1 aggressor screening protocol. Traditional CSDS and our published CNSDS parameters state that aggressors must attack the intruders within 1 min (Golden et al. 2011). However, we and others have found that inclusion of female mice may delay initial attacks from the CD-1, resulting in many CD-1 mice not reaching the threshold to be considered an aggressor despite eventually attacking both male and female intruders within the five-minute screening period. We show that latency to attack the male and female intruder does not correlate to aggression displayed during CNSDS, while the number of attacks across the three days of screening is a better predictor of overall aggression. CD-1 mice that attacked an average of 1 or more times during screening attacked at least 3 times during CNSDS (Fig. 1D). Therefore, we defined a threshold of 1 attack per screening session on average to predict aggressor behavior. CD-1 mice that do not meet the threshold should be categorized as non-aggressors, while CD-1 mice that meet or surpass the threshold should be categorized as aggressors.

A second aim of this study was to further investigate CNSDS as a valid chronic stress paradigm in both male and female C57BL/6J mice. Our initial report of CNSDS and additional data from other groups using or adapting CNSDS suggest that CD-1 aggressors attack male intruders significantly more than females (Zhu et al. 2023; Smith et al. 2023; Bennett et al. 2024). We show that despite receiving fewer attacks (Fig. 2D), female mice show similar susceptibility to CNSDS as male mice (Fig. 3) when using a new approach to categorization resilience and susceptibility. This new approach compares time spent interacting with a novel CD-1 mouse and time spent interacting with a novel C57 of the opposite-sex, which specifically assesses social avoidance of the aggressor. Decreased social interaction is a common behavioral phenotype observed after social defeat stress (Krishnan et al. 2007); however, some studies report typical social behavior in female mice in response to CNSDS (Parel et al. 2023; Schuler et al. 2024). Other studies have investigated alternative methods of measuring susceptibility to chronic stress in female mice, such as close vigilance (Willmore et al. 2022), change in velocity (Schuler et al. 2024), and novelty-suppressed feeding (Parel et al. 2023). While highly useful and informative, these measures do not allow for direct comparison between male and female subjects.

Importantly, by defining the social interaction ratio as time spent interacting with a novel CD-1 mouse compared to time spent interacting with a C57 mouse of the opposite sex, we can investigate specific avoidance of the stressor rather than general social avoidance. Traditional chronic social defeat stress paradigms investigate social avoidance by comparing time spent interacting with a novel CD-1 mouse to time spent without a mouse present, typically resulting in a 60/40 split of SUS and RES mice respectively (Golden et al. 2011). When using the traditional SIT ratio after CNSDS, we do not see the expected split of SUS and RES mice (Fig. 3). CNSDS utilizes pairs of experimental C57 male and female mice that experience social defeat sessions together over the ten days. This is a different experience for experimental C57 mice than traditional social defeat stress that only uses male mice. Because subjects are exposed to multiple mice during stress, it is important to distinguish between social avoidance of the CD-1 aggressor and avoidance of opposite sex C57 mice. By introducing a novel C57 mouse in the social interaction test, we can more specifically investigate social avoidance of a CD-1 aggressor and utilize this to measure susceptibility to the stress. Future experimentation with this new SIT ratio is necessary for broader validation. Specifically, future experiments should compare the new SIT ratio to the traditional ratio in a variety of behavioral tasks after exposure to CNSDS, including the open field, elevated plus maze, novelty suppressed feeding, and sucrose preference. This new SIT ratio may address limitations of the traditional ratio in accurately assessing stress susceptibility in female mice.

Despite receiving fewer attacks than males, female mice show susceptibility to the CNSDS procedure. One potential explanation is that, in addition to direct physical attacks, there may be an additional component of vicarious, or witness, stress experienced by the female intruders. Vicarious social defeat stress is a model in which a mouse witnesses the social defeat of another mouse, without being physically attacked by the aggressor, and can lead to effects such as increased avoidance behaviors, increased corticosterone levels, and decreased sucrose preference (Sial et al. 2016; Iñiguez et al. 2018; Schuler et al. 2024). A recent study by Schuler et al. (2024) investigated both witness social defeat stress and CNSDS and reported that SUS males display decreased SIT ratios while SUS females display reduced changes in velocity. These similarities observed by Schuler et al. in females across CNSDS and witness defeat stress could suggest that there are similar stress components or experiences across both procedures.

A third aim of this study was to investigate whether CNSDS-induced susceptible and resilient phenotypes correlated with reward valuation behavior. Importantly, reward valuation behavior is highly conserved across species (Der-Avakian et al. 2016), and abnormal reward valuation is observed in major depressive disorder (Rupprechter et al. 2021). Therefore, assessing chronic stress effects on reward valuation has a high translational value. Chronic stress disrupts brain systems involved in reward processing, leading to altered decision making and diminished sensitivity to rewards (Ironside et al. 2018). We previously reported that chronic stress results in the abolishment of sensitivity to satiety-based outcome devaluation, leading to similar responding in the valued and devalued trials (Dieterich et al. 2021). Importantly, this is the first study to investigate whether susceptible and resilient phenotypes to chronic stress correlate with sensitivity to outcome devaluation. Resilient mice pressed significantly more in the valued task compared to the devalued task, while susceptible mice pressed less in both valued and devalued trials (Fig. 4). This suggests that while susceptible mice have an abolished or decreased sensitivity to satiety-based outcome devaluation (as expected with chronic stress), resilient mice are more sensitive. One potential explanation is an overall decrease of responding specifically in the susceptible group. Therefore, it is possible that susceptible mice are less likely to expend effort in an operant task (Dieterich et al. 2021). A potential future experiment is to perform a progressive ratio or concurrent choice operant task after mice are exposed to CNSDS and SIT to determine if susceptible mice show a decreased breakpoint and less lever pressing than resilient mice. Another future experiment could be to determine if stress susceptibility impacts motivation behaviors that are affected by CNSDS, such as effort-related choice (Dieterich et al. 2021). Mice categorized into different stress susceptibility groups may show changes in willingness to exert effort, which could support our findings from the outcome devaluation task.

Importantly, the CNSDS paradigm enables inclusion of females in chronic social defeat stress experiments and allows potential analysis of sex differences in behavioral tasks. Although we did not observe any sex differences in the current study and therefore combined male and female groups for analysis (Shansky 2019), previous research has reported such differences. Previously we reported that CNSDS exposure eliminated outcome devaluation in females and reduced sensitivity in males (Dieterich et al. 2021). A limitation of this study is the underpowered sample sizes of males (*n* = 5) and females (*n* = 8) across SUS and RES groups, which limited our ability to analyze sex differences. Future studies should include larger and balanced male and female cohorts to better assess potential sex differences.

When screening CD-1 mice as aggressors, it is important to note that the degree of aggressive behavior varies greatly. Approximately half of all screened CD-1 mice will not reach the criteria for inclusion (Golden et al. 2011), which means many CD-1 mice will not be used in the experiment. During sensory exposure co-housing, intruder and CD-1 mice are permitted limited sensory interaction (such as smell and sight), but physical contact is prevented. Social avoidance of CD-1 mice following chronic social defeat stress is likely due to conditioned fear learning, which is extinguishable following 16 days of sensory-only exposure to aggressors (Ayash et al. 2020). Additionally, 12 days of physical exposure to non-aggressive CD-1 mice decreased freezing behavior and increased SIT ratio in male intruder mice (J. Kim and Kim 2025). In CNSDS, either the male or female C57BL/6J mouse are co-housed with the CD-1 aggressor with which they had interacted, while the other is co-housed with a novel CD-1. We have previously had success using non-aggressors as these novel CD1 mice for sensory exposure co-housing to help limit the number of CD-1 mice required for CNSDS (Dieterich et al. 2021). However, it is possible that five days (out of ten) of sensory exposure co-housing with non-aggressors may in part extinguish the CD1 fear. Further research is necessary to determine if this is the case.

There is a growing need for preclinical research using female mice to study translationally relevant behaviors for mood disorders. Our updated CNSDS paradigm will facilitate the inclusion of female mice in preclinical chronic stress research and better permit direct comparisons between males and females. CNSDS is effective in both sexes and allows increased inclusion of females in the preclinical study of neurobiological circuits involved in mood disorders such as MDD. In addition, CNSDS allows for investigation of the mechanisms underlying stress susceptibility and resilience in both sexes. Ultimately, CNSDS should allow for the study of whether new therapeutic interventions for mood disorders may be effective in both men and women.

## Data Availability

We will share and present all data that conforms to quality standards with sufficient experimental detail to allow researchers to reproduce and extend upon our peer-reviewed and archived work.
